# Experimental Design Modeling of the Effect of Hexagonal Wurtzite—ZnO Synthesis Conditions on Its Characteristics and Performance as a Cationic and Anionic Adsorbent

**DOI:** 10.3390/molecules24213884

**Published:** 2019-10-28

**Authors:** Mai M. Khalaf, Enshirah Da’na, Kawther Al-Amer, Manal Hessien

**Affiliations:** 1 Department of Chemistry, King Faisal University, P.O. Box 400, Alahsa 31982, Saudi Arabia; mmkali@kfu.edu.sa (M.M.K.); kalamer@kfu.edu.sa (K.A.-A.); 2 Chemistry Department, Faculty of Science, Sohag University, Sohag 82524, Egypt

**Keywords:** nanoparticles, adsorption, surface composite design, ZnO, methyl orange, methylene blue

## Abstract

Surface composite design was used to study the effect of the ZnO synthesis conditions on its adsorption of methyl orange (MO) and methylene blue (MB). The ZnO was prepared via hydrothermal treatment under different conditions including temperature (T), precursor concentration (C), pH, and reaction time (t). Models were built using four Design expert-11 software-based responses: the point of zero charge (pHzc), MO and MB removal efficiencies (R_MO_, R_MB_), MO and MB adsorption capacities (q_MO_, q_MB_), and hydrodynamic diameter of ZnO particles (D_h_). ZnO was characterized by X-ray diffraction (XRD), Fourier-transform infrared (FTIR) spectroscopy, UV/VIS spectroscopy, thermal gravimetric analysis (TGA), and dynamic light scattering (DLS). The formation of ZnO was confirmed by the XRD, UV, and FTIR spectra. Results showed a very high efficiency for most of the samples for adsorption of MB, and more than 90% removal efficiency was achieved by 8 samples among 33 samples. For MO, more than 90% removal efficiency was achieved by 2 samples among 33 samples. Overall, 26 of 31 samples showed higher MB adsorption capacity than that of MO. R_MB_ was found to depend only on the synthesis temperature while R_MO_ depends on temperature, pH, and reaction time. pHzc was found to be affected by the synthesis pH only while D_h_ depends on the synthesis pH and precursor concentration.

## 1. Introduction

Metal oxides have attracted huge attention recently due to their important applications in many fields. Among them, the ZnO has received great attention due to attractive characteristics including its novelty, diversity, and easily controlled morphologies [1], which can be used in a wide range of applications such as drug delivery and anticancer [2], antimicrobial [3,4], photocatalysis [1,5,6,7], Light-emitting diode (LEDs) [8], environmental applications [8,9], solar cells [7,10], drugs, cosmetics, antibacterial coatings for fabrics, and antibacterial packaging for food [11].

These characteristics make ZnO a very attractive candidate for exploring new applications. However, development of ZnO for new applications requires the ability to control morphological structures within reasonable synthetic conditions, which is one of the biggest challenges [10]. So far, many different ZnO structures such as flowers [1,12], needles [12], wires [13], rods [14,15,16], spheres [17,18], and stars [3,19] have been reported [1]. In addition to morphological structure, it is important for some applications to be able to control band gap energy for photocatalytic applications [17], particle size distribution [17,20,21], and zero point charge [22] for anionic and cationic adsorption applications.

Several ZnO synthesis routes have been described in the literature with different morphologies and excellent characteristics, including chemical precipitation [6], solvothermal [19], sol–gel processing [7], thermal decomposition [15], microwave irradiation [5], chemical bath deposition [4], dip coating [23], spin coating [20], electrical deposition [23], and hydrothermal processing [1,12,13,18,24,25,26]. Among these methods, hydrothermal processing has been proven to be a flexible approach to ZnO synthesis due to its simplicity, ease, and relatively low fabrication cost [11,24]. Furthermore, it offers a path for homogeneous nucleation with high purity without the need for calcination.

Many factors have been reported to affect the properties of the synthesized ZnO via hydrothermal processing such as synthesis temperature [5,16,25,26,27], precursor concentration [6,19], pH of synthesis mixture [12,13,25,26], and reaction time [28]. Understanding the effects of all of these factors by changing only one variable each time requires a large number of experiments, long time, and high cost. Furthermore, it is impossible to draw trustworthy conclusions regarding interactions between these factors following this research routine [24].

Design of experiments (DOE) offers the ability to improve ZnO characteristics, and reduces process variability, time, the number of experiments, and cost. Furthermore, DOE is very powerful in determining the extent of interactions among the studied variables [25], although, to our best knowledge, DOE methodology has very limited publications on ZnO applications [26,29,30] and synthesis [31,32].

The toxicity and carcinogenic nature of dyes pose a risk to the environment. Furthermore, their degradation may lead to the production of carcinogenic compounds such as p-aminoazobenzene [33]. Accordingly, huge interest has been put in the development and synthesis of ZnO nanoparticles for removal of dyes such as methylene blue (MB) and methyl orange (MO). However, most of the reported work was dedicated to the development of ZnO for photocatalytic degradation of these dyes [9,26,33,34,35,36]. On the other hand, very limited reports can be found about the adsorption removal of these dyes, even though it is more advantageous over the degradation because of its low cost, simple design, and no formation of harmful by-products [29,37,38,39,40,41,42].

In our previously published work [43], one-dimensional nanorods, two-dimensional nanoflakes, and nanoplates ZnO were synthesized via a hydrothermal process. It was found that the growth and the morphology of nano ZnO were strongly affected by Zn concentration, pH, temperature, and growth time. All the synthesized samples were characterized with Fourier-transform infrared (FTIR) spectroscopy, X-ray diffraction (XRD) analysis, and scanning electron microscopy (SEM). In this study, further investigation of the effects of these factors on the point of zero charge (pH_ZC_), hydrodynamic diameter (D_h_), and the cationic and anionic adsorption performance of the ZnO following the central composite design methodology. This statistical methodology can be applied to correlate a specific response to synthesis conditions in order to minimize process variability, cost, the number of experiments, and time. Furthermore, it is a very helpful tool in determining the magnitude of interactions among the major effects [30].

## 2. Materials and Methods

### 2.1. Materials

Zinc nitrate (Zn(NO_3_)_2_), Methyl orange (C_14_H_14_N_3_SO_3_Na, C.I.13025), MB (C_16_H_18_ClN_3_S), and potassium hydroxide (KOH) were bought from Sigma-Aldrich. All the primary chemicals used in this work were of analytical grade.

### 2.2. Preparation of Zinc Hydroxide

A specific amount of Zn(NO_3_)_2_ was weighed according to experimental design, shown in Table 1 and Table 2, and then dissolved in 40 mL of distilled water. The mixture was stirred at 40 rpm to ensure complete salt dissolving. The mixture pH was then adjusted by adding 1 M KOH solution dropwise at a rate of 1 mL/min via a burette. The pH of the solution was monitored using an Orion 2 Star pH meter until the required pH was reached according to Table 1 and Table 2.

### 2.3. Hydrothermal Treatment

The Zn(OH)_2_ precipitate was transferred from the beaker to a Teflon container, sealed very well in an autoclave and then heated in an oven at the required temperature (T) and for required time according to Table 1 and Table 2.

### 2.4. Washing and Drying

After the hydrothermal treatment, the sample was transferred from the autoclave into a beaker and the liquor (supernatant) was poured out. Then, the sample was sonicated in 50 mL of distilled water for 90 min using an ultrasonic system (Powersonic 405). After that, the sample was left to settle overnight, the liquor was then poured, and 20 mL of ethanol was added. The sample was again sonicated for 90 min and then left to settle overnight. The liquor was poured again and the sample was left in the oven overnight at 60 °C to get rid of any retained water. Finally, the samples were collected and coded according to Table 2 and kept until being used.

### 2.5. Methyl Orange and Methylene Blue Solution Preparation

Forty milligrams of MO or MB were dissolved in 1 L of double distilled water to prepare a 40 ppm stock solution of each. These two solutions were consecutively diluted to prepare different concentrations in a range of 5–30 ppm. Only the 20 ppm solution was used for the adsorption tests, and the other concentrations were used for the calibration of the UV instrument before each test. HNO_3_ or NaOH was used to control the pH of the solutions by an Orion 2 Star pH meter.

### 2.6. Characterization

A high-resolution transmission electron microscope (JEOL JEM-1011 Transmission Electron Microscope) was used for TEM imaging. The Cary 630 FTIR spectrophotometer was used to collect the FTIR spectra of the ZnO samples. UV/VIS absorption was performed with the Shimadzu UV-1800 spectrophotometer. The Cilas Nano DS dual light scattering particle size analyzer was used to perform dynamic light scattering (DLS). Prior to UV and DLS, ZnO samples were sonicated in deionized, double distilled water using Powersonic 405 for 1.5 h at 20 °C. Structural properties of ZnO samples were investigated via XRD using a Burker D8 Advance XRD system with a Ni-filled Cu-Kα radiation at a wavelength of 1.54060 Å. ZnO nanoparticles were scanned at a rate of 2.0°/min from 10° to 60°. Thermal gravimetric analysis (TGA) was performed to investigate the thermal stability of the synthesized ZnO using a TA instrument model SDT Q600. Twenty milligrams of the ZnO sample was heated at a rate of 2 °C/min from 25 to 1000 °C in an inert medium. The Brunauer–Emmett–Teller (BET) analysis of two selected samples 30 and 34 (best and worst adsorbents) were measured by N2 adsorption at 77 K using a BET surface area analyzer (Micromeritics ASAP 2020). Prior to analysis, samples were degased at 300 °C for 350 min of holding time to remove the impurities from the surface of the samples.

### 2.7. Point of Zero Charge

The pH_ZC_ of the synthesized ZnO was determined following the procedure reported elsewhere [44]: 8 beakers, each containing 20 mL of 0.1 M NaCl solution. The initial pH (pH_i_) of each beaker was adjusted at a certain value within a range of 2–11 using HNO_3_ and NaOH with an Orion 2 Star pH meter. Then, 10 mg of the ZnO sample were stirred in each beaker for 24 h at 120 rpm and 293 K. Finally, samples were filtered and the final pH (pH_e_) was determined.

### 2.8. Adsorption Tests

MO and MB adsorption was performed by mixing 0.025 g of ZnO with 10 mL of 20-ppm solution for 24 h at 293 K and 140 rpm. The MB pH was controlled at 4.5 using HNO_3_, while MO solution pH was 8.0 without adjustment. The residual MO and MB in the solution were detected with the Shimadzu UV-1800 spectrophotometer at λ_max_ of 464 nm for MO and 664 nm for MB, and the following equation was used to calculate the removal efficiency:

$$R=\;\frac{C_{i}-C_{e}}{C_{i}}\times 100,$$

where C_i_ and C_e_ are the initial and equilibrium concentrations of dye in ppm, respectively.

It is worth mentioning that the models presented in Table 3 are assuming a constant variance and time independence. Consequently, experimental runs randomization was essential. The ZnO synthesis conditions and their levels are shown in Table 1 as coded and real values, while Table 2 shows the detailed experimental runs with their random run order and all the measured responses.

Accordingly, it was important to test that the models have met these assumptions via residual plots. Residuals are the differences between the fitted and the observed responses. For model adequacy, it is important for any measured response to have a normal distribution of residuals probability. Moreover, for model adequacy, the residual versus the run order plot should not reveal any pattern.

## 3. Results and Discussion

**Figure 1:**
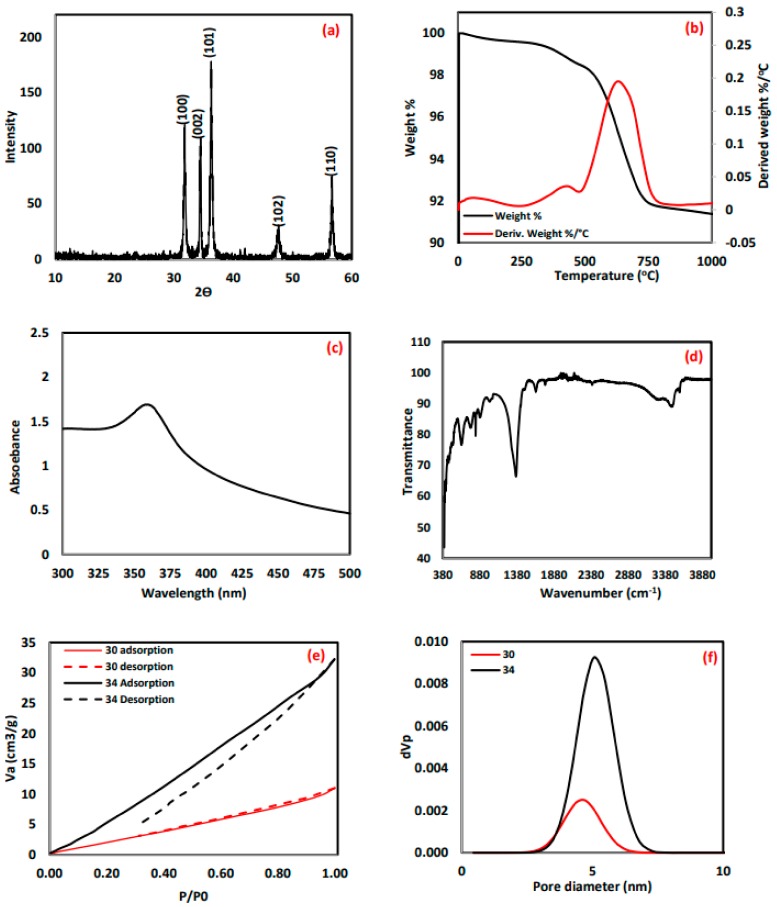
(**a**) XRD pattern of sample 31 prepared under the conditions of C = 0.2 M, pH = 7, T = 100 °C, and t = 1 h. (**b**) Thermal gravimetric analysis (TGA) and DTA analysis results of sample 10 prepared under the conditions of C = 0.4 M, pH = 7, T = 100 °C, and t = 2 h. (**c**) UV-VIS spectrum of sample 1 prepared under the conditions of C = 0.3 M, pH = 11, T = 150 °C, and t = 1.5 h. (**d**) FTIR spectrum for the range of 4000–400 cm^−1^ of sample 1 prepared under the conditions of C = 0.2 M, pH = 7, T = 100 °C, and t = 1 h. (**e**) Nitrogen adsorption desorption isotherms for samples 30 and 34. (**f**) Pore size distributions for samples 30 and 34.

The XRD pattern of a representative sample of the synthesized ZnO nanoparticles is shown in Figure 1a. The diffraction peaks at 2θ values of 31.73°, 34.44°, 36.21°, 47.49°, and 56.67° corresponding to miller indices of 100, 002, 101, 102, and 110, respectively, confirm the formation of hexagonal wurtzite phase of ZnO according to the standard JCPDS 80-0075. All the synthesized ZnO samples have a similar pattern and are available at our previously published work [43].

The TGA and DTA analyses (Figure 1b) revealed that the ZnO lost about 1% of its weight when the temperature reached 104 °C, which may be related to the desorption of CO_2_ and H_2_O attached to the surface, and 2% weight loss of the total weight occurred at about 130 °C, which is related to strongly bonded water. The maximum rate of weight loss was 0.2%/min, at which 8% loss of material occurred at around 692 °C. The exothermic peak at 692 °C in the DTA curve is a result of the decomposition of traces of Zn(OH)_2_ existing in the sample [45]. These results reveal a good thermal stability of the sample.

Ten milligrams of each ZnO sample were added to 10 mL of deionized water and then sonicated using Powersonic 405. The solution was then used to perform the UV-VIS measurement. ZnO formation was confirmed by the appearance of absorption bands in a range of 358–380 nm, as shown in Figure 1c, for a representative sample (spectra of the rest of the samples are available in Figure S1 in Supplementary Materials). Bodke et al. reported peaks at 363 and 361 nm [46]. Khorsand et al. and Liang et al. recorded a characteristic absorption peak of ZnO at a wavelength of 375 nm [17,21]. Ibupoto et al. reported absorption peaks of ZnO at wavelengths of 382 and 385 nm [27]. Ali et al. reported a peak at 380 nm [5]. Andrade et al. [3] reported ZnO with hexagonal wurtzite phase with absorption bands in a range of 424–433 nm. Anandan et al. [6] reported absorption spectra of ZnO synthesized under different concentrations and found that the precursor concentration has an important effect on the adsorption characteristics. The absorption band they reported was in a range of 364–380 nm, which is a characteristic of the ZnO hexagonal phase. All these reported results confirmed that the samples prepared in this work are all ZnO with good crystallinity of the ZnO with minimal impurity [5,6].

The FTIR spectra of all the prepared samples were recorded in the range from 4000 to 400 cm^−1^ to detect the functional groups, confirming the existence of ZnO. All samples exhibited a similar spectra as presented in Figure 1d (spectra of other samples are available in Figure S2 in Supplementary Materials), which shows a band at 823 cm^−1^ due to hydrogen-bonded O–H stretch. In addition, the band at 1640 cm^−1^ resulted from the CO_2_ adsorbed on the ZnO surface during storage [23]. Absorption bands at 1050 and 3480 cm^−1^ related to CO_2_ (C–O) and H_2_O (O–H) adsorbed on the surface [21,33,34]. The sharp, intense peaks at 420 and 406 cm^−1^ are related to the ZnO which appeared in all samples, confirming that the prepared samples were ZnO [3,5,6]. Furthermore, ZnO stretching absorption was detected in all samples at around 642 cm^−1^ and 887 cm^−1^ [46].

The nitrogen adsorption isotherms for one of the best adsorbent (sample 30) and one of the worst adsorbent (sample 34) are presented in Figure 1e and the pore size distributions of the two samples are shown in Figure 1f. It is worth mentioning that complex porous materials may reveal structures from multiple isotherm types according to its pore characteristics [47]. Sample 34 shows an open hysteresis with the desorption curve lower than the adsorption curve, which is most likely due to one of the following reasons: (i) the presence of ırregular microspores or bottle-shaped pores; (ii) a porous defect of the porous sample; (iii) slow penetration of the N_2_ molecules into very narrow pores, resulting in non-establishment of thermodynamic equilibration on any part of the adsorption–desorption isotherm; and (iv) pore blocking or cavitation associated [47,48].

Sample 30 exhibited a surface area of 13.8 m^2^g^−1^ and a pore size of 4.83 nm, while sample 34 exhibited a higher surface area of 51.6 m^2^g^−1^ but a smaller pore size of 3.81 nm. The differences of surface areas and pore size are most likely associated with the crystalline structure of the two samples. For large molecules like dyes, the size of a molecule may control the diffusion in the bulk and within the pores of the adsorbent [47]. If the pore size of the adsorbent is close to the size of the adsorbate molecule, the adsorption process will be controlled by diffusion within the pores. Accordingly, the pore size of the adsorbent become more important than the surface area, since the majority of the surface area are located inside the pores, which means that if the molecules cannot diffuse inside the pores this surface area will not be available for adsorption. This explains why sample 30 has better performance than sample 34, even if it has a lower surface area.

**Figure 2:**
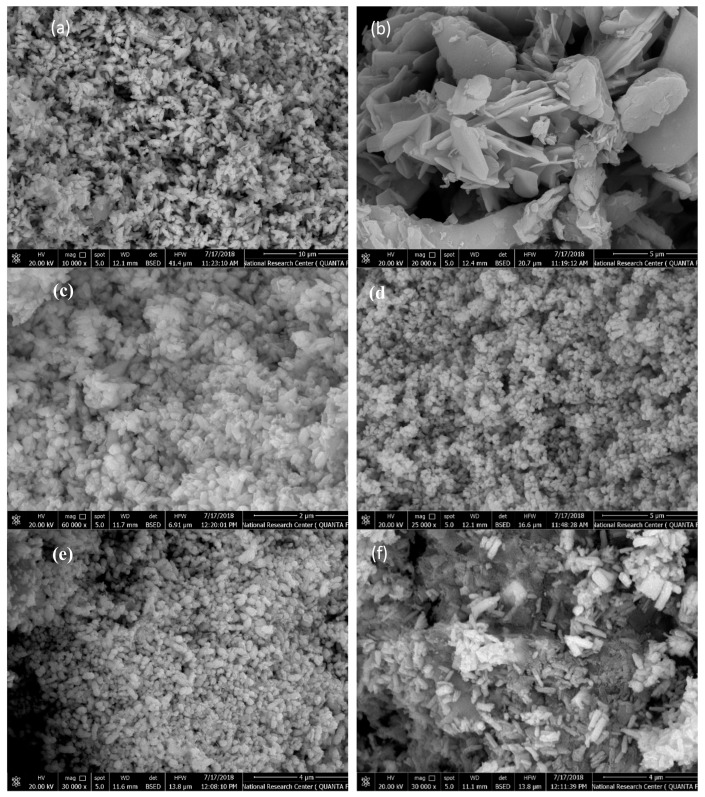

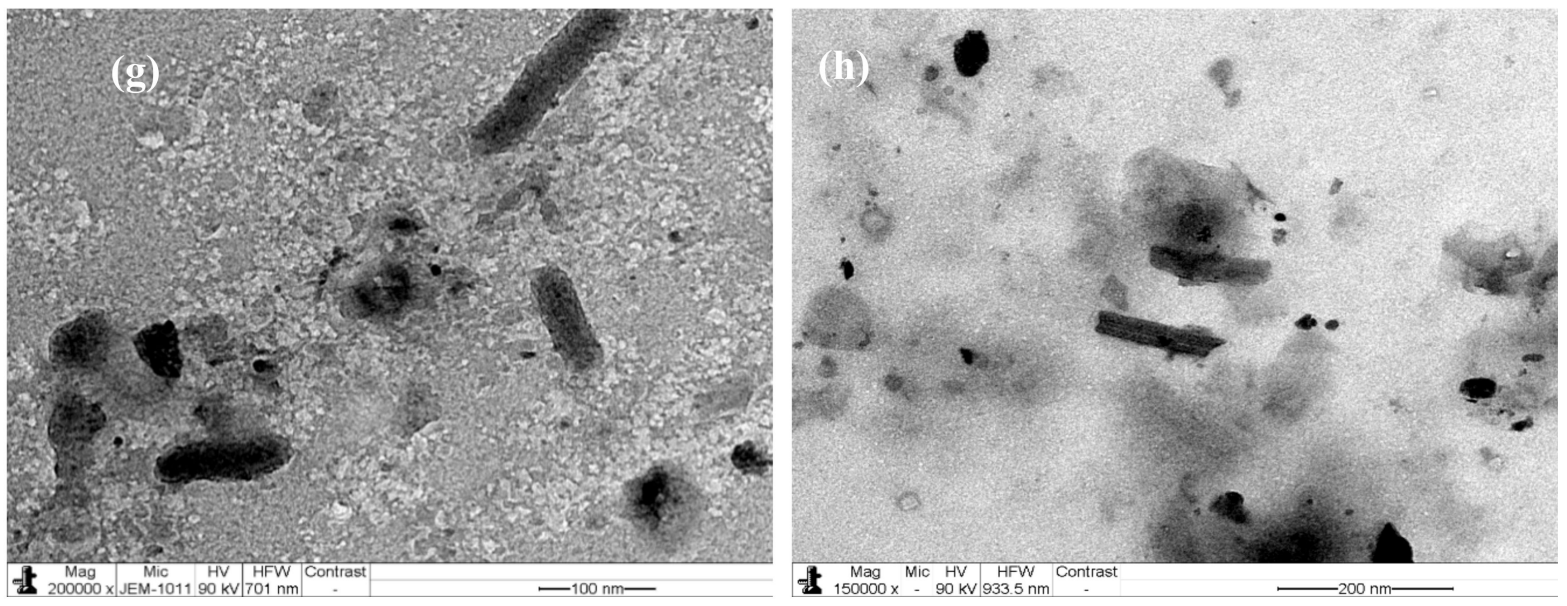
FE-SEM images of ZnO samples prepared under different conditions: (**a**) C= 0.1 M, pH = 9, T = 150 °C, and t = 1.5 h; (**b**) C= 0.5 M, pH = 9, T = 150 °C, and t = 1.5 h; (**c**) C= 0.3 M, pH = 11, T = 150 °C, and t = 1.5 h; (**d**) C = 0.3M, pH = 9, T = 50 °C, and t = 1.5 h; (**e**) C = 0.3 M, pH = 9, T = 150 °C, and t = 1.5 h; (**f**) C = 0.3 M, pH = 7, T = 150 °C, and t = 1.5 h. (**g**) TEM images of ZnO sample prepared under the conditions of C = 0.3 M, pH = 9, T = 150 °C, and t = 1.5 h; (**h**) TEM images of ZnO sample prepared under the conditions of C = 0.3 M, pH = 9, T = 150 °C, and t = 1.5 h.

Figure 2 displays the SEM and TEM images of some ZnO samples prepared under different conditions. It is obvious that different morphologies such as short nanorods, elliposoidal rods, irregular nanoflakes, and nanoplates with wide and narrow pore size distributions were obtained under different synthesis conditions. Figure 2d,e, for example, shows the effect of fabrication temperature. At 50 °C (Figure 2d), nanoplates were obtained. Increasing the temperature to 150 °C changes the morphology into shorter nanorods (Figure 2e), as shown in TEM images Figure 2g,h. Figure 2a,b,e show the effect of the Zn precursor concentration. Short nanorods were observed at 0.1 M (Figure 2a). Increasing the concentration to 0.3 M increased the diameter of the obtained nanorods (Figure 2e). At 0.5 M concentration, both nanorods and nanoflakes were formed. Figure 2c,e,f show the effect of synthesis pH. At pH = 7 (Figure 2f), nanorods were formed. By increasing the pH to 9 (Figure 2e), nanoplates and short nanorods were formed. At pH = 11 (Figure 2c), an ellipsoidal shape with tapered tops were formed. More detailed discussion about the effect of synthesis conditions on the morphology of ZnO can be found in our previous contribution [43].

**Figure 3:**
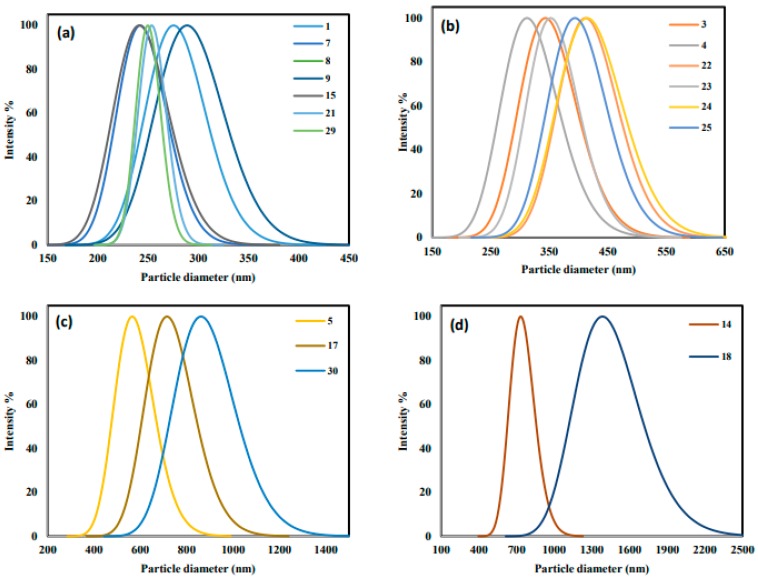
ZnO particle size distribution obtained from dynamic light scattering (DLS) for samples 1, 7, 8, 9, 15, 21, and 21 (**a**), samples 3, 4, and 22–25 (**b**), samples 5, 7, and 30 (**c**), samples 8 and 14 (**d**). Details of each sample synthesis conditions can be found in Table 2.

The D_h_ of the ZnO obtained by the DLS method is shown in Figure 3. The results show that the D_h_ of the ZnO prepared varies within a range from 218 to 8920 nm.

ZnO nanoparticles synthesis generally involves nucleation and crystal growth. Let us start with the formation of nuclei unit (Zn(OH)_2_) and the growth unit ([Zn(OH)_4_]^2−^, according to the following equations [49,50]:

(1) $$Zn^{2+}+2OH^{-}↔Zn{\left(OH\right)}_{2}\;,$$

(2) $$Zn^{2+}+4OH^{-}↔{\left[Zn{\left(OH\right)}_{4}\right]}^{2-}\;,$$

(3) $${\left[Zn{\left(OH\right)}_{4}\right]}^{2-}↔\;Zn{\left(OH\right)}_{2}+2OH^{-},$$

(4) $$Zn{\left(OH\right)}_{2}↔\;ZnO+H_{2}O,$$

(5) $${\left[Zn{\left(OH\right)}_{4}\right]}^{2-}↔\;ZnO+H_{2}O+2OH^{-}\;.$$

The D_h_ of the synthesized ZnO is controlled by both nucleation and crystal growth [51,52]. According to Equations (2) and (3), Zn(OH)_2_ and [Zn(OH)_4_]^2−^ coexist in equilibrium. When Zn(OH)_2_ nuclei exist in a large amount and the growth unit [Zn(OH)_4_]^2−^ exists in a small amount, the nucleation rate will be higher than the crystal growth rate. Accordingly, relatively small particles are obtained. Conversely, when the growth unit [Zn(OH)_4_]^2−^ exists in a large amount and Zn(OH)_2_ nuclei exists in a small amount, the nucleation rate will be slower than the crystal growth rate, and thus, relatively large particles are obtained [52].

The equilibrium between Zn(OH)_2_ and [Zn(OH)_4_]^2−^ is controlled by the synthesis conditions, such as the temperature, the pH, and the OH:Zn^2+^ ratio. Zn(OH)_2_ in the solution dissolves to Zn^2+^ and OH^–^ till the concentrations of Zn^2+^ and OH^−^ reach a certain critical values, and then crystallization of ZnO from the solution occurs and begin to grow, according to Equations (4) and (5). Since ZnO is much less soluble than Zn(OH)_2_, Zn(OH)_2_ tends to convert into ZnO [49].

Increasing synthesis temperature increases ZnO solubility and thus results in dissolving crystals. As a result, small crystals disappear from the solution while the large ones slightly reduced in size. Accordingly, smaller particle size distributions may be obtained by increasing the hydrothermal temperature [49].

A similar range of particle size was obtained by changing the time from 0.5 to 2.5 h. However, changing the concentration from 0.1 to 0.5 M resulted in a particle size within a range from 430 nm to 1250 nm. Similarly, changing pH within a range of 7–11 had a great impact on the particle size with a varied range of 375–850 nm. As discussed earlier, the Zn(OH)_2_:[Zn(OH)_4_]^2−^ ratio is strongly affected by the pH and OH:Zn^2+^ ratio. Accordingly, both nucleation and growth rates are affected by C and pH. However, the model shown in Table 3 indicates that the D_h_ is only significantly affected by the precursor concentration and the synthesis pH and that the effects of both temperature and time are negligible.

The F-value of 6.60 (Table 3) indicated a significant model with only a 0.48% chance that this value could occur due to noise. A lack of fit F-value of 78.23 suggests there is a 8.91% chance that this value is due to noise. The adjusted R² value of 0.2858 is in good agreement with the predicted R² value of 0.1332 with a difference less than 0.2.

**Figure 4:**
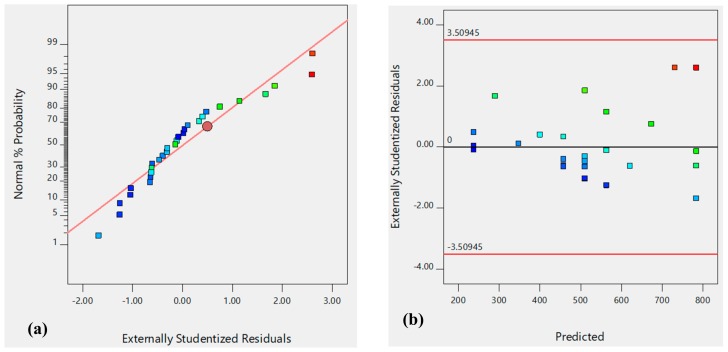
Model adequacy tests for D_h_ response: Normal probability of the externally studentized residuals (**a**), Predicted versus externally studentized residuals (**b**).

To further ensure the model adequacy, residual plots were prepared and are shown in Figure 4. The model reliability can be confirmed, as the residual plot follows a normal distribution (Figure 4a) with a structure of less distribution when plotted against predicted values (Figure 4b).

The obtained pH_ZC_ values of all samples are within a range of 5.9–8.1, as shown in Table 2. It is clear that the ZnO surface stoichiometry was affected by the synthesis conditions and even the ZnO surface purity and water content could be affected [53]. This was also detected by the FTIR analysis as CO_2_ and H_2_O bands appeared in some samples and did not appear in others (FTIR analysis for all samples can be found in our previous contribution) [43].

**Figure 5:**
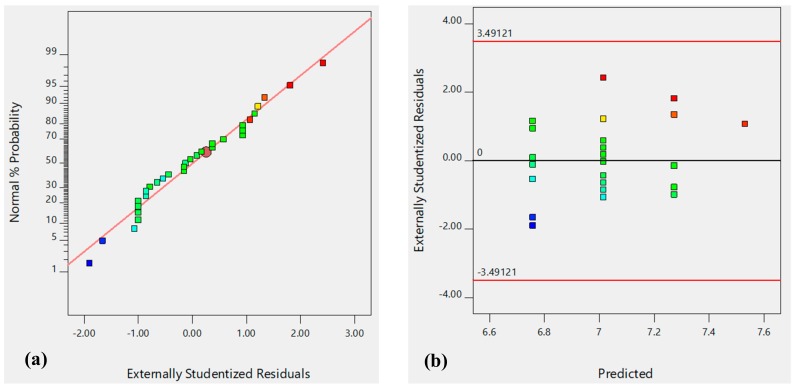
Model adequacy tests for pH_ZC_ response: Normal probability of the externally studentized residuals (**a**), Predicted versus externally studentized residuals (**b**).

Table 2 shows the pH_ZC_ obtained for samples prepared under different conditions. Increasing temperature from 50 to 250 °C increased the pHzc of ZnO samples from 7.1 to 8.1. The pHzc value of 8.1 corresponds to a partially or totally dehydrated surface. An increase in the pHzc of the ZnO samples from 7.2 to 8.1 was achieved by increasing the synthetic pH value from 7 to 11. This could be related to the effect of pH on the species ionization state, and accordingly, different species could be adsorbed on the ZnO surface. A very limited effect of precursor concentration and reaction time on the pH_ZC_ was observed. Probably, the differences in pH_ZC_ in this study resulted from the formation or adsorption of Zn hydrolytic complexes on the ZnO surface changing the surface stoichiometry [53]. According to the models shown in Table 3, synthesis pH has a significant effect on the pH_ZC_ within the 95% confidence interval. This result is confirmed by the residual plots shown in Figure 5, with a normal probability plot (Figure 5a) and a plot of residuals with random distribution against predicted values (Figure 5b).

**Figure 6:**
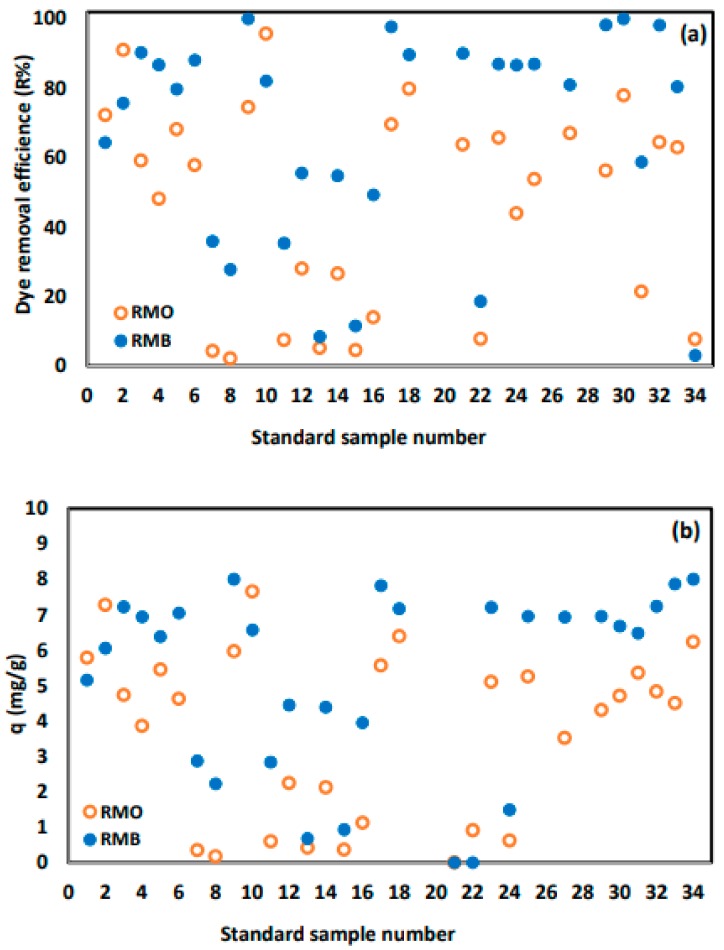
(**a**) Removal efficiencies (R) and (**b**) adsorption capacities (q) of MO and MB by adsorption on the 34 prepared ZnO samples.

To examine the MO and MB removal efficiencies, a set of experiments was performed under the same conditions using different sample each times. It can be seen from Figure 6 that, for most of the samples, MB removal efficiency was higher than that of MO considering the formula weights of the MB = 319.9 g/mol and of the MO = 327.3 g/mol, and MB with a smaller weight had a better diffusion rate than MO. Thus, there is an inverse relationship between the molecular weight of the dye molecules and their adsorption rates from aqueous media. Furthermore, it is very clear to observe the effect of synthesis conditions on the adsorption performance, as 100% removal efficiency was achieved by some samples such as sample 9 and sample 30 while other samples recorded 0% removal efficiency such as sample 20. Interestingly, the samples with high adsorption efficiency were able to effectively remove both cationic (MB) and anionic (MB) dyes. On the other hand, samples with weak performance were weak toward both of the dyes as well. This is very attractive as one adsorbent can be used to adsorb cations and anions by simply controlling the surface charge of the adsorbent. The pH_ZC_ of the samples were within a range of 5.9–8.1. Thus, when the samples are to adsorb MB, it should be used in a basic medium, i.e., pH > pH_ZC_. At such a pH, the surface of all samples will be bearing a negative charge. Accordingly, it will attract the positively charged dye. In the case of adsorbing MO, an acidic medium, i.e., pH < pH_ZC_, is required to ensure positively charged samples to be able to attract the negative dye.

Usually, for comparison with other adsorbents, it is more useful to study adsorption capacity of the adsorbent. Thus, similar to the removal efficiency, the adsorption capacities of all samples were calculated (Table 2) and the results are presented in Figure 6b. It is very clear that q and R have the same trend for both MB and MO since all experiments were performed under similar conditions of initial concentration, mass of adsorbent, and volume of solution. As shown in Table 2, a maximum MB adsorption capacity of 8 mg/g was achieved with samples 9 and 30, while, for MO, a maximum adsorption capacity of 7.66 mg/g was achieved using sample 10.

Table 3 clearly shows that the models obtained for q_MB_ and q_MO_ are affected by the same factors as the models obtained for R_MB_ and R_MO_. The main factor affecting the adsorption capacity of MB is temperature, while, for MO, temperature, pH, and reaction time are important factors. This result is confirmed by the residual plots shown in Figure S4, with a normal probability plot and a plot of residuals with random distribution against predicted values.

Table 4 summarizes some contributions in the literature for the adsorption of MB and MO on ZnO. It is not easy to compare the results obtained because of the variation of adsorption conditions used. However, the results obtained in this work are very attractive, as they show a high removal efficiency (100%) at a low concentration, implying that samples 10 and 29 are very promising for such an application.

The models given in Table 3 show that R_MB_ depends on the synthesis temperature within the 95% confidence interval used, while R_MO_ depends on temperature, pH, and reaction time.

**Figure 7:**
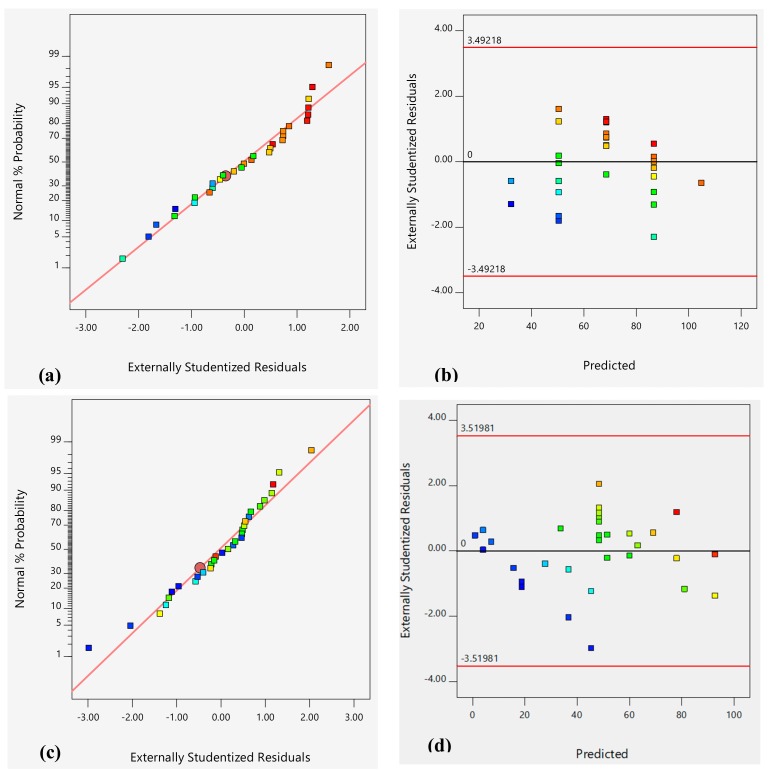
Model adequacy tests for R_MO_ response: Normal probability of the externally studentized residuals (**a**), Predicted versus externally studentized residuals (**b**). Model adequacy tests for R_MB_ response: Normal probability of the externally studentized residuals (**c**), Predicted versus externally studentized residuals (**d**).

The respective R_MO_ and R_MB_ F values of 23.3 and 14.77 (Table 3) imply that the models are significant with a chance less than 0.1% and these values are large due to noise. Furthermore, for both models, the residuals F values are 5.91 and 7.338 for MO and MB, respectively, with *P*-values higher than 5%, confirming that residuals are 15.48% for MO and 10.94% for MB due to noise, confirming the model adequacy. Residuals plots are shown in Figure 7a,b for R_MB_ and in Figure 7c,d for R_MO_. There is no doubt that the models are correct since the normal probability plots are linear and the plots of residuals against predicted values do not follow any pattern.

## 4. Conclusions

ZnO nanoparticles were synthesized via a hydrothermal technique under different conditions following surface composite design methodology. The main responses tested within the 95% confidence interval were the ability of the synthesized ZnO to adsorb the MO cationic dye and the MB anionic dye from aqueous solutions, the pH_ZC_ of the adsorbent, and the average particle size of ZnO. The ZnO formation was confirmed by the XRD, UV-VIS, and FTIR spectra via the appearance of the ZnO characteristic peaks. Adsorption results showed an excellent performance of some samples with a removal efficiency of up to 100% for MB and a maximum removal efficiency of 96% for MO, which demonstrated that an excellent candidate can be provided for removal of both dyes. R_MO_ shows a dependency on T, pH, and t, while R_MB_ depends only on T. The estimated D_h_ obtained by DLS varied within a range of 218–8920 nm because of changing synthesis conditions. According to the model, the D_h_ was affected mostly by the substrate concentration and synthesis pH. A wide range of pH_ZC_ (5.9–8.1) was obtained for the samples, which gives the samples a diverse range of adsorption applications for both cationic and anionic species. The pH_ZC_ model indicated the dependency on synthesis pH at the 95% confidence interval used.

## Figures and Tables

**Table 1:** Independent variables and their levels used for the central composite design.

Variable	Symbol	Level in Coded and Real Values		
		−1	0	+1
Precursor concentration (M)	C	0.2	0.3	0.4
Temperature (°C)	T	100	150	200
Time (h)	t	1	1.5	2
pH	pH	7	9	11

**Table 2:** Independent variables and their real values and responses measured.

Standard	Random	C (M)	pH	T (°C)	Time (h)	Point of Zero Charge (pH_ZC_)	D_h_ (nm)	Adsorption Capacity of MB (q_MB;_ mg/g)	Adsorption Capacity of MO (q_MO;_ mg/g)	Removal Efficiency of MO (R_MO_; %)	Removal Efficiency of MB (R_MB_; %)
1	27	0.2	7	100	1	7.3	279	5.15	5.78	72.30	64.41
2	6	0.4	7	100	1	5.9	1322	6.06	7.28	91.00	75.72
3	8	0.2	11	100	1	6.8	350	7.22	4.74	59.20	90.26
4	23	0.4	11	100	1	6.8	318	6.94	3.86	48.20	86.77
5	30	0.2	7	200	1	6.7	537	6.38	5.46	68.20	79.76
6	5	0.4	7	200	1	6	642	7.05	4.63	57.86	88.15
7	20	0.2	11	200	1	6.9	242	2.87	0.35	4.37	35.92
8	26	0.4	11	200	1	6.8	311	2.23	0.17	2.18	27.83
9	28	0.2	7	100	2	6.5	280	8.00	5.96	74.56	100.00
10	7	0.4	7	100	2	7.2	412	6.57	7.66	95.75	82.09
11	12	0.2	11	100	2	7.2	218	2.83	0.60	7.52	35.38
12	1	0.4	11	100	2	7.9	536	4.45	2.24	28.05	55.60
13	10	0.2	7	200	2	7.2	824	0.68	0.42	5.20	8.51
14	14	0.4	7	200	2	6.8	752	4.39	2.13	26.59	54.83
15	2	0.2	11	200	2	6.8	247	0.93	0.37	4.60	11.58
16	4	0.4	11	200	2	7.2	366	3.94	1.12	14.01	49.29
17	33	0.1	9	150	1.5	7.3	647	7.82	5.57	69.59	97.74
18	24	0.5	9	150	1.5	7.2	1252	7.17	6.39	79.87	89.65
19	29	0.3	5	150	1.5	No precipitation was observed					
20	25	0.3	13	150	1.5	8	8920	0.00	0.91	11.39	0.00
21	11	0.3	9	50	1.5	6.7	260	7.21	5.11	63.83	90.10
22	9	0.3	9	250	1.5	8.1	435	1.49	0.62	7.80	18.61
23	34	0.3	9	150	0.5	6.8	352	6.96	5.26	65.72	86.94
24	13	0.3	9	150	2.5	7.6	262	6.93	3.52	44.03	86.69
25	18	0.3	9	150	1.5	6.6	397	6.96	4.31	53.86	86.98
26	22	0.3	9	150	1.5	6.6	462	6.68	4.71	58.92	83.45
27	32	0.3	9	150	1.5	7.2	1475	6.48	5.36	67.06	80.98
28	21	0.3	9	150	1.5	6.6	417	7.24	4.83	60.43	90.54
29	15	0.3	9	150	1.5	7.1	437	7.86	4.51	56.32	98.30
30	3	0.3	7	150	1.5	7.2	851	8.00	6.24	77.95	100.00
31	19	0.3	11	150	1.5	8.1	373	4.70	1.71	21.41	58.74
32	16	0.4	9	150	1.5	6.9	472	7.86	5.16	64.48	98.19
33	17	0.2	9	150	1.5	7	497	6.44	5.03	62.93	80.49
34	31	0.3	9	250	2.5	6.5	937	0.24	0.62	7.76	3.05

**Table 3:** The point of zero charge (pHzc), methyl orange (MO) and methylene blue (MB) removal efficiencies (R_MO_ and R_MB_, respectively), MO and MB adsorption capacities (q_MO_ and q_MB_, respectively), and hydrodynamic diameter of ZnO particles (D_h_) and their models summary.

**D_h_**	**R-sq**	**R-sq (adj)**	**R-sq (pred)**	**F-value**	***P*-value**	**Residual F**	**Residual P**
	0.3369	0.2858	0.1332	6.60	0.0048	78.23	0.0891
Uncoded equation:		D_h_ = +913.86185 + 1102.69231C − 81.56481pH					
Coded equation:	D_h_ = +510.59 + 110.27C − 163.13pH						
**pH_ZC_**	**R-sq**	**R-sq (adj)**	**R-sq (pred)**	**F-value**	***P*-value**	**Residual F**	**Residual P**
	0.1711	0.1425	0.0450	5.99	0.0207	2.45	0.3313
Uncoded equation:		pH_ZC_ = +5.85612 + 0.128835pH					
Coded equation:	pH_ZC_ = +7.02 + 0.2577pH						
**R_MO_**	**R-sq**	**R-sq (adj)**	**R-sq (pred)**	**F-value**	***P*-value**	**Residual F**	**Residual P**
	0.7331	0.6920	0.6410	23.30	<0.0001	5.91	0.1548
Uncoded equation:		R_MO_ = +212.48271 − 10.319pH − 0.326843T − 14.75094t					
Coded equation:	R_MO_ = +48.46 − 20.64pH − 16.34T − 7.38t						
**R_MB_**	**R-sq**	**R-sq (adj)**	**R-sq (pred)**	**F-value**	**P-value**	**Residual F**	**Residual P**
	0.3453	0.2478	0.3220	14.77	0.0006	7.338.59	0.1094
Uncoded equation:	R_MB_ = +123.11199 − 0.363235T						
Coded equation:	R_MB_ = +68.63 − 18.16T						
**q_MO_**	**R-sq**	**R-sq (adj)**	**R-sq (pred)**	**F-value**	***P*-value**	**Residual F**	**Residual P**
	0.7185	0.6847	0.6301	21.27	<0.0001	93.15	0.0817
Uncoded equation:	q_MO_ = +17.08333 − 0.84444pH − 0.026065T − 1.17318t						
Coded equation:	q_MO_ = +3.81 − 1.69pH − 1.3T − 0.5866t						
**q_MB_**	**R-sq**	**R-sq (adj)**	**R-sq (pred)**	**F-value**	***P*-value**	**Residual F**	**Residual P**
	0.3467	0.3226	0.2482	14.33	0.0008	10.45	0.2404
Uncoded equation:	q_MB_ = +9.80868 − 0.029016T						
Coded equation:	q_MB_ = +5.46 − 1.45T						

* Detailed model equations and calculations can be found in Supplementary Materials.

**Table 4:** Adsorption of MB and MO by ZnO reported in the literature.

Adsorbate	Adsorption Conditions	q (mg/g)	Reference
MB	pH = 6, m = 0.5 g, initial concentration (Ci) = 40 ppm	34.19	[37]
MB	V/m = 0.625 L/g, Ci = 50 ppm	27.45	[38]
MB	V/m = 10 L/g, pH = 6, Ci = 100 ppm	198	[39]
MB	V/m = 1 L/g, pH = 6, Ci = 10 mmol/L	7 mmol/g	[40]
MB	V/m = 0.5 L/g, Ci = 16 ppm	7.67	[41]
MO	V/m = 2 L/g, pH = 6.5, Ci = 50 ppm	11	[42]
MB	V/m = 0.4 L/g, pH = 8, Ci = 20 ppm	7.86	Current work (sample 29)
MO	V/m = 0.4 L/g, pH = 4.5, Ci = 20 ppm	7.66	Current work (sample 10)

## Supplementary Materials

The following are available online at https://www.mdpi.com/1420-3049/24/21/3884/s1.
