# Supplementary material for: Experimental Design Modeling of the Effect of Hexagonal Wurtzite—ZnO Synthesis Conditions on Its Characteristics and Performance as a Cationic and Anionic Adsorbent
Source: Molecules. 2019 Oct 28;24(21):3884. doi: 10.3390/molecules24213884 (PMC6864852; doi:10.3390/molecules24213884)
Supplement: Supplementary file 1 [file molecules-24-03884-s001.pdf]

Experimental design modeling of the effect of hexagonal wurtzite - ZnO synthesis conditions on its characteristics and performance as a cationic and anionic adsorbent

Mai M. Khalaf[1,2], Enshirah Da'na[1*], Kawther Al-Amer[1] and Manal Hessien[1*]

[1]Department of Chemistry, King Faisal University, Alahsa, 31982, P.O. Box 400, Saudi Arabia
[2]Chemistry Department, Faculty of Science, Sohag University, Sohag 82524, Egypt
* Correspondence: edana@kfu.edu.sa; mhessien@kfu.edu.sa Telephone: 00966135897540; Fax: 00966135899557

Supplementary materials

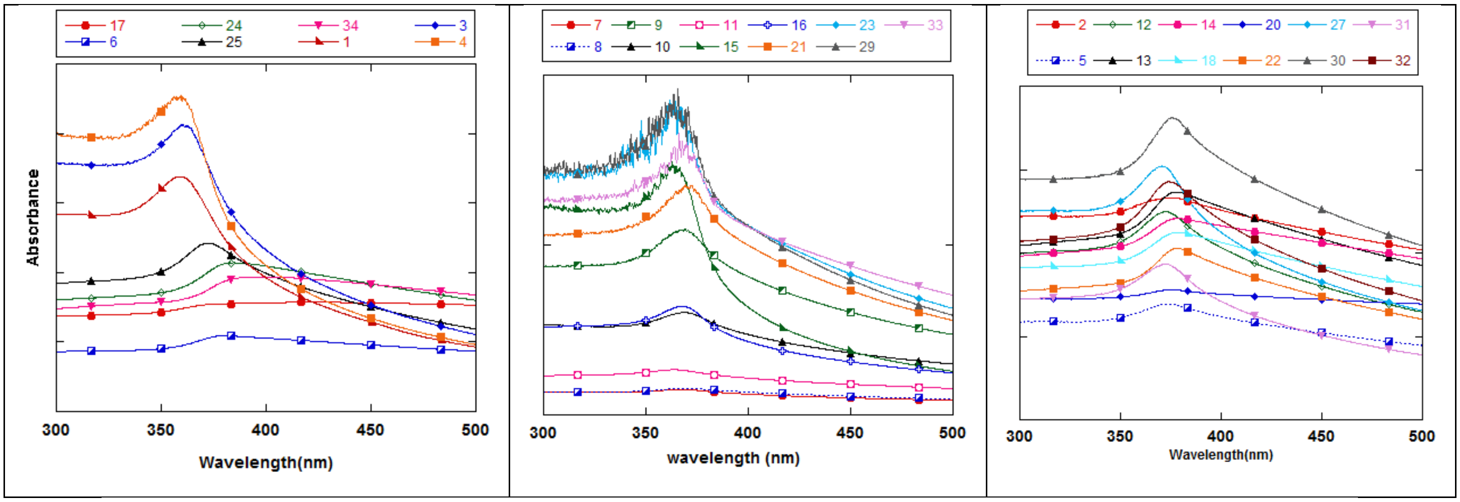

Fig. S1. UV-Vis spectrum of ZnO samples.

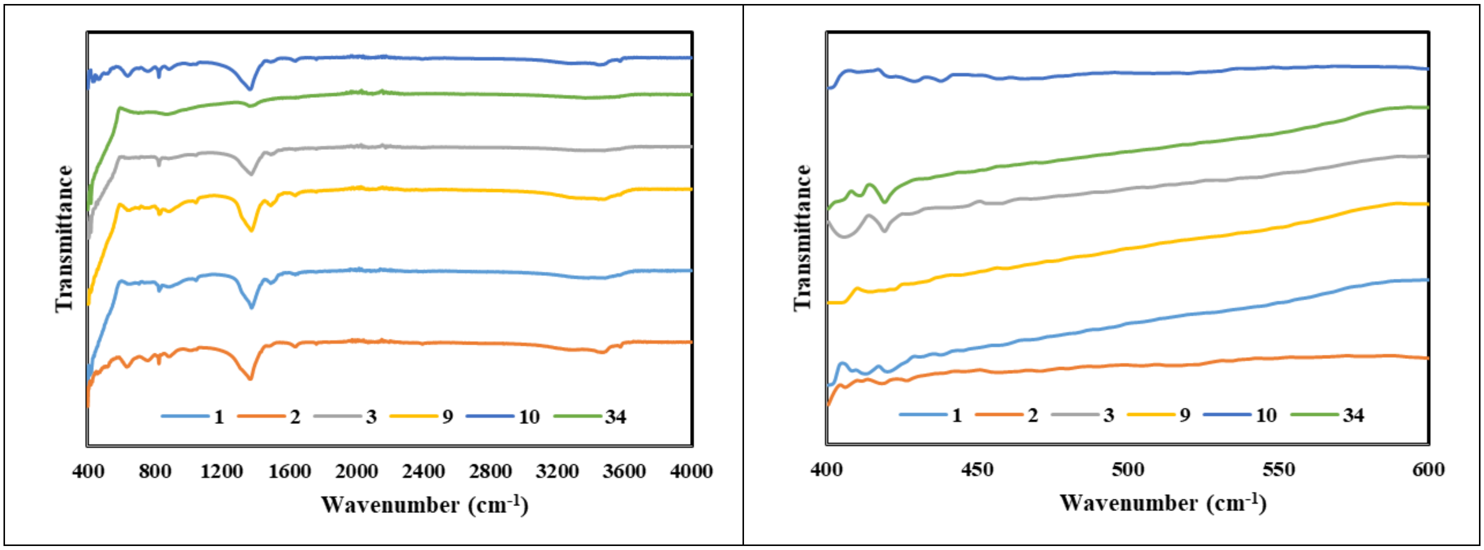

Fig. S2. FTIR spectrum of ZnO samples 1, 2, 3, 9, 10, and 34 for the range 4000-400 cm^{-1}(a) and 600-400 cm^{-1} (b).

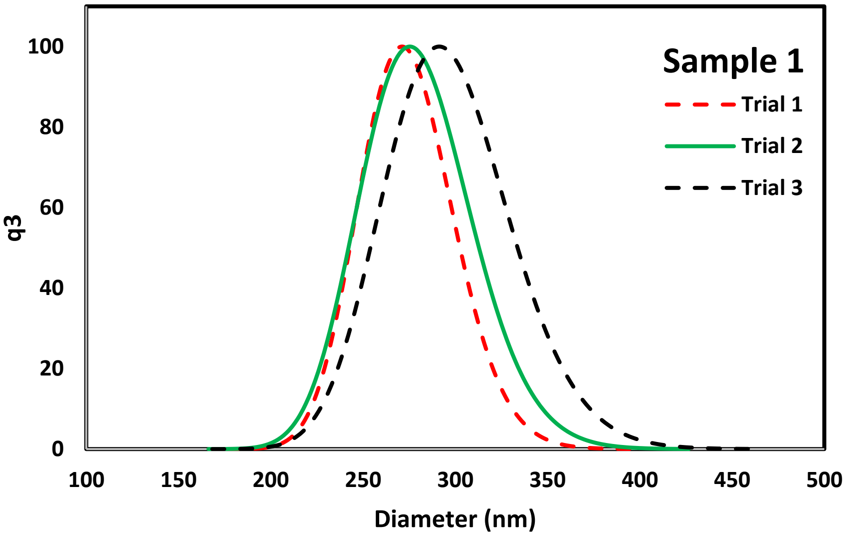

D1(nm)	272.2
D2(nm)	275.4
D3(nm)	290.1
Average D (nm)	279.23
Standard deviation	9.52

Fig. S3: The triplicate measurements of Dh for sample 1 with average and standard deviation shown.

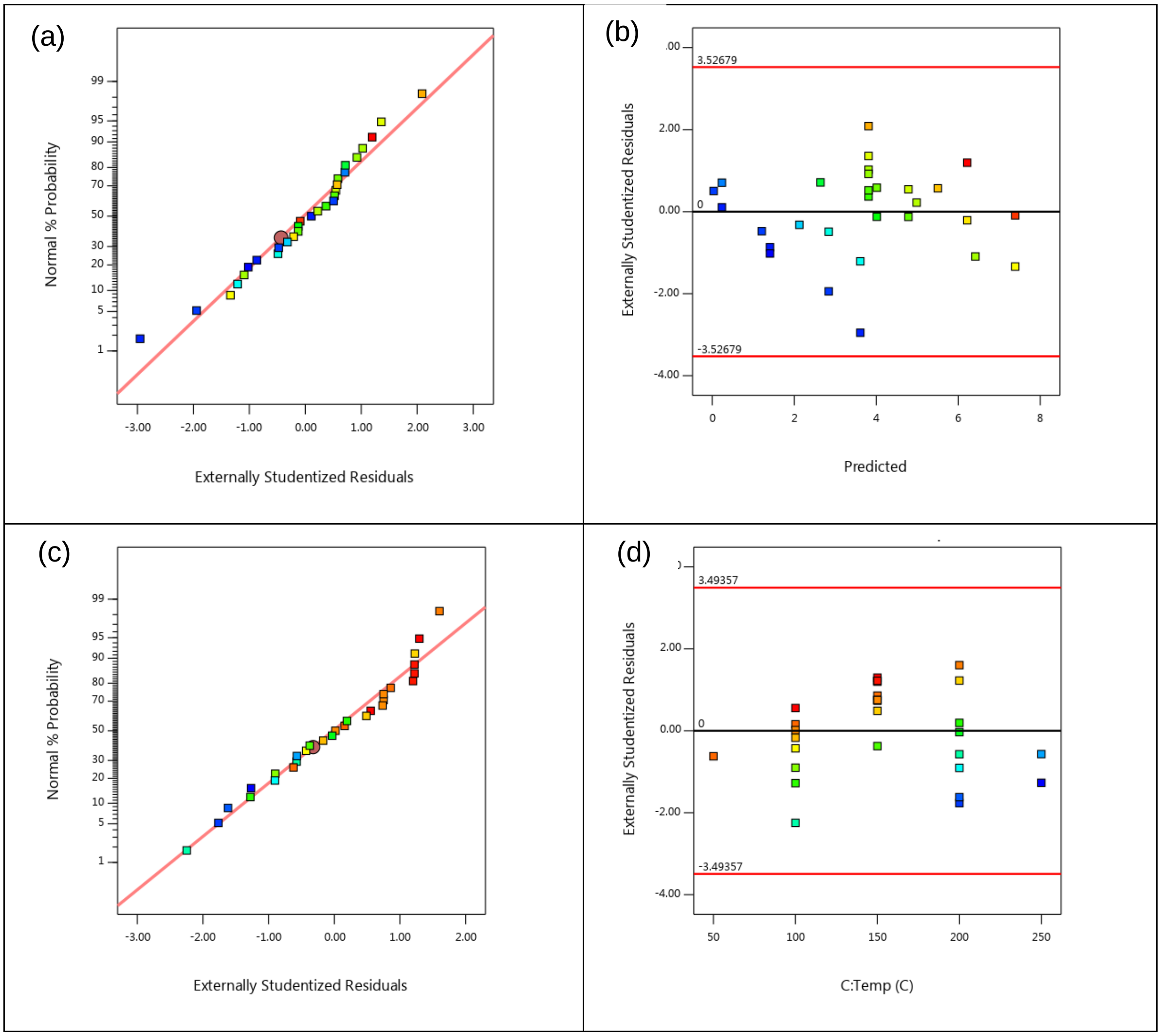

Fig S4. Model adequacy tests for q_{MO} (a and b) and q_{MB} (c and d) responses.

Model details

Design of Experiments

When process factors (independent variables) satisfy an important assumption that they are measurable, continuous, and controllable by experiments, with negligible errors, the procedure was carried out as follows:

1) A series of experiments were performed for adequate and reliable measurement of the response of interest.

2) A mathematical model of the second-order response surface with the best fit was developed.
3) The direct and interactive effects of the process parameters (factors) were statistically tested to determine which effect are important.

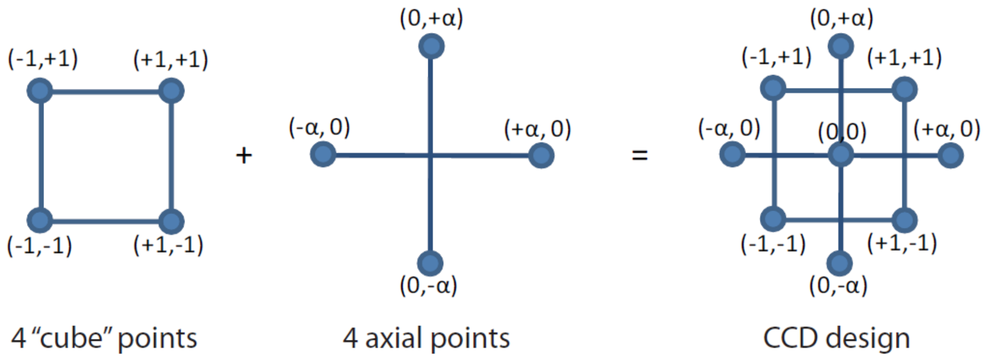

Central composite design (CCD) design: 4 "cube" points, 4 axial points, and 5 replicates at the center point (0,0).

Once the desired value ranges of the variables had been defined, they were coded to lie at ±1 for the factorial points, 0 for the center points, and ±α for the axial points. The codes were calculated as functions of the range of interest of each factor, as shown in the table below.

Code	Actual value of factor
$-\alpha$	$X_{\min}$
-1	$\frac{(\alpha-1)X_{\max}+(\alpha+1)X_{\min}}{2\alpha}$
0	$\frac{X_{\max}+X_{\min}}{2}$
$+1$	$\frac{(\alpha-1)X_{\min}+(\alpha+1)X_{\max}}{2\alpha}$
$+\alpha$	$X_{\max}$

$X_{\max}$ and $X_{\min}$: maximum and minimum value of X.

The factors used in this study are shown in the following diagram. The first one shows screen shot from the deign expert program clarifying the number of samples with respect to concentration and pH and the second figure shows number of samples with respect to temperature and time.

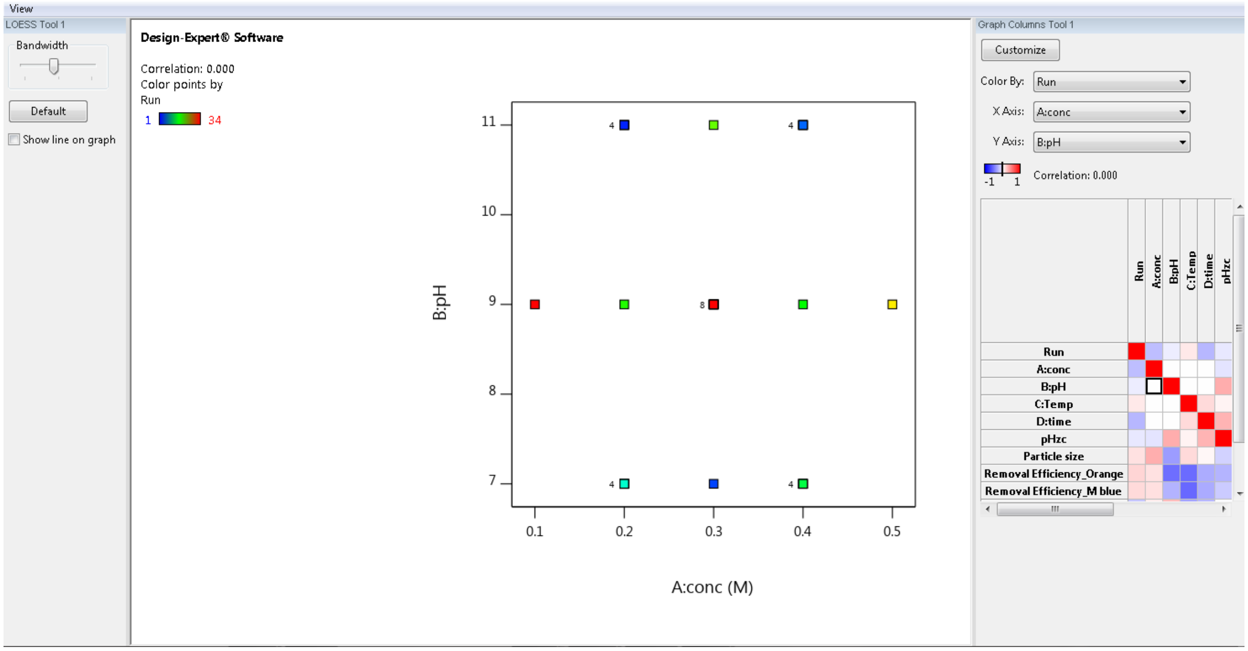

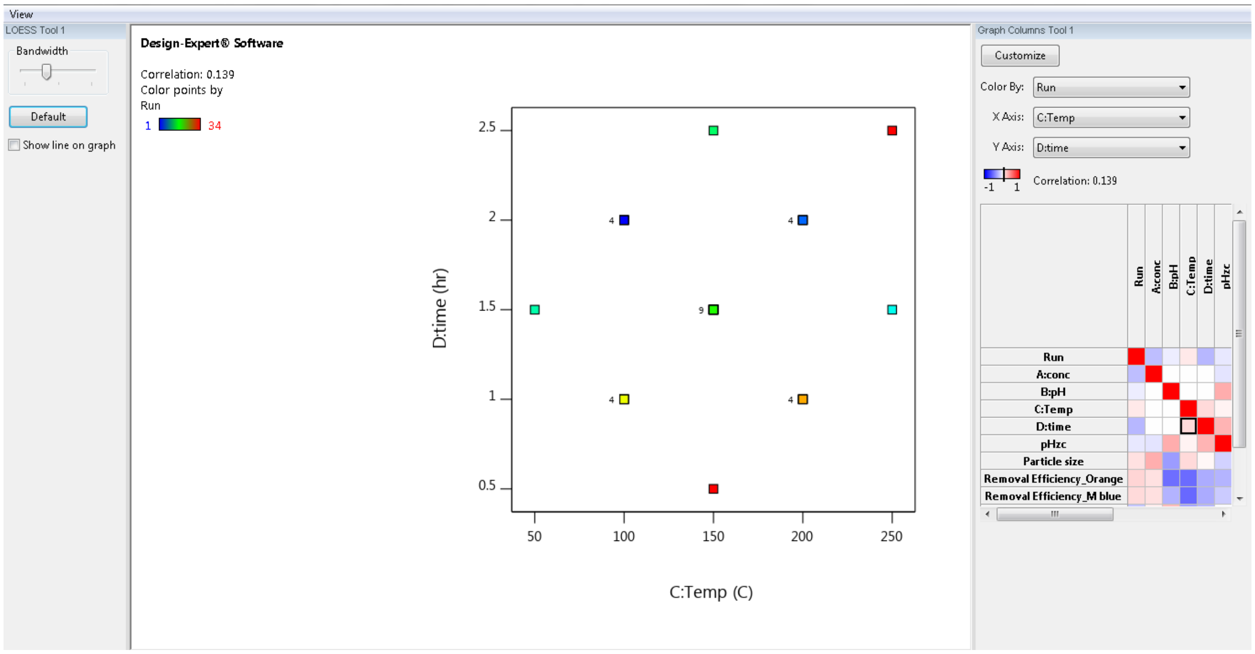

The responses can be expressed as second-order polynomial equations, according to the following equation:

$$Y = f(x) = \beta_0 + \sum_{i=1}^{k} \beta_i x_i + \sum_{i=1}^{k} \sum_{j=i+1}^{k} \beta_{ij} x_i x_j + \sum_{i=1}^{k} \beta_{ii} x_i^2$$

where Y is the predicted response (Dh, R_{MB}, R_{MO}, or pH_{ZC}) used as a dependent variable; k the number of independent variables (factors), xi (i = 1, 2) the input predictors or controlling variables (factors); β0 the constant coefficient, and βi, βij and βii the coefficients of linear, interaction and quadratic term, respectively. The coefficient parameters were estimated using a multiple linear regression analysis employing the software Design-Expert (version 11).

The models were then checked using a numerical method employing the coefficient of determination (R^2), adjusted R^2 (R^2_{adj}), and then calculated as shown in below. R^2 indicates how much of the observed variability in the data was accounted for by the model, while R^2_{adj} modifies R^2 by taking into account the number of covariates or predictors in the model.

$$R^2 = 1 - \frac{SS_{\text{residual}}}{SS_{\text{model}} + SS_{\text{residual}}}$$

$$R_{\text{adj}}^2 = 1 - \frac{n-1}{n-p}(1 - R^2)$$

where SS is the sum of the squares, n the number of experiments, and p the number of predictors (term) in the model, not counting the constant term. Furthermore, an R^2_{adj} close to the R^2 values insures a satisfactory adjustment of the quadratic models to the experimental data. Therefore, the regression models explained the removal efficiency well.

The figures below show the error parameters obtained for one of the models as an example.

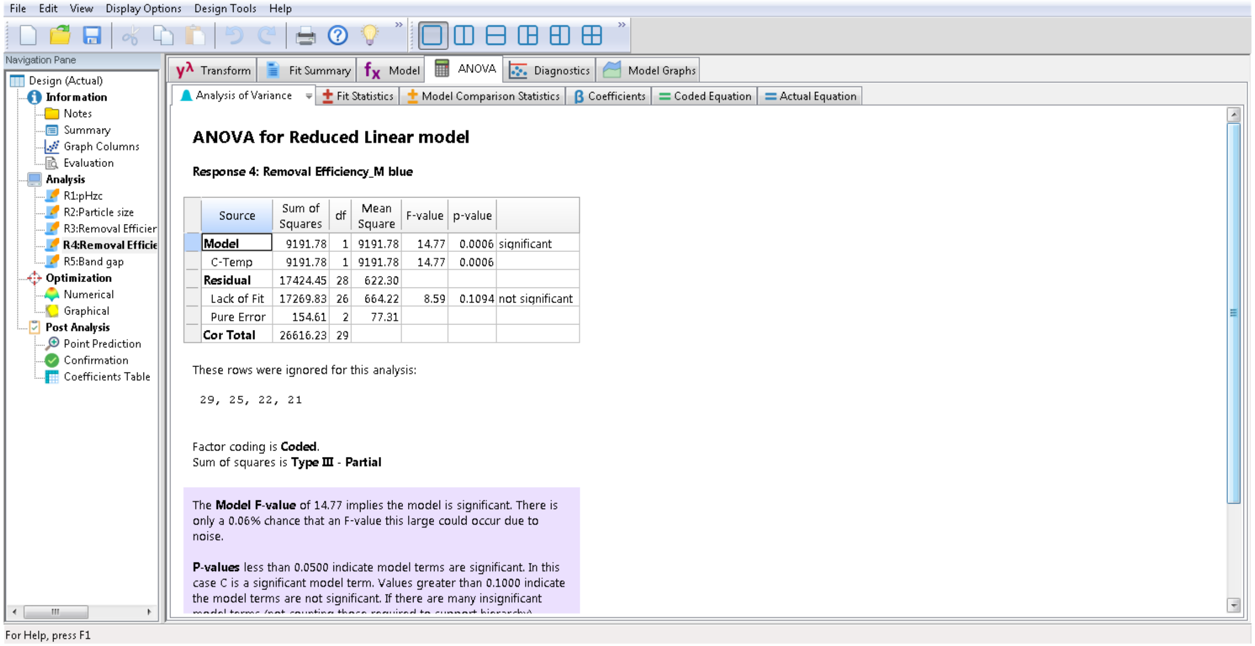
File Edit View Display Options Design Tools Help
Navigation Pane
Design (Actual)
Information
Notes
Summary
Graph Columns
Evaluation
Analysis
R1:pHzc
R2:Particle size
R3:Removal Efficier
R4:Removal Efficie
R5:Band gap
Optimization
Numerical
Graphical
Post Analysis
Point Prediction
Confirmation
Coefficients Table
Transform
Fit Summary
Model
ANOVA
Diagnostics
Model Graphs
Analysis of Variance
Fit Statistics
Model Comparison Statistics
Coefficients
Coded Equation
Actual Equation
ANOVA for Reduced Linear model
Response 4: Removal Efficiency_M blue
Source | Sum of Squares | df | Mean Square | F-value | p-value
Model | 9191.78 | 1 | 9191.78 | 14.77 | 0.0006 | significant
C-Temp | 9191.78 | 1 | 9191.78 | 14.77 | 0.0006
Residual | 17424.45 | 28 | 622.30
Lack of Fit | 17269.83 | 26 | 664.22 | 8.59 | 0.1094 | not significant
Pure Error | 154.61 | 2 | 77.31
Cor Total | 26616.23 | 29
These rows were ignored for this analysis:
29, 25, 22, 21
Factor coding is Coded.
Sum of squares is Type III - Partial
The Model F-value of 14.77 implies the model is significant. There is only a 0.06% chance that an F-value this large could occur due to noise.
P-values less than 0.0500 indicate model terms are significant. In this case C is a significant model term. Values greater than 0.1000 indicate the model terms are not significant. If there are many insignificant
For Help, press F1

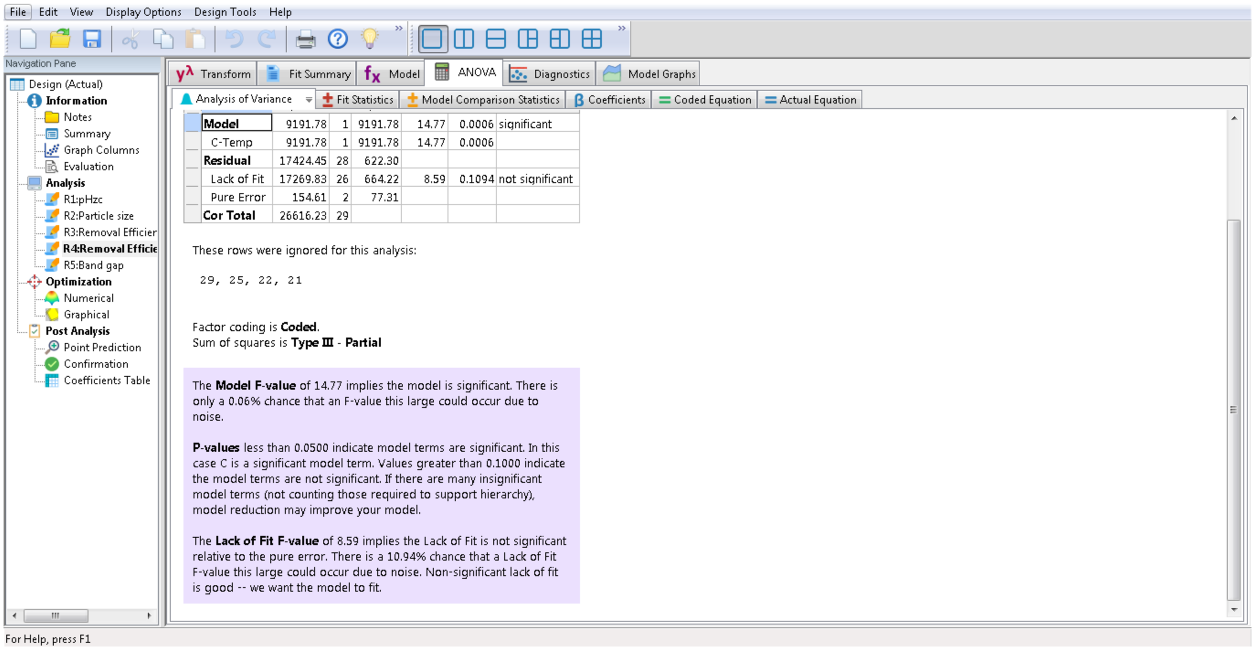
File Edit View Display Options Design Tools Help
Navigation Pane
Design (Actual)
Information
Notes
Summary
Graph Columns
Evaluation
Analysis
R1:pHzc
R2:Particle size
R3:Removal Efficier
R4:Removal Efficie
R5:Band gap
Optimization
Numerical
Graphical
Post Analysis
Point Prediction
Confirmation
Coefficients Table
Transform
Fit Summary
Model
ANOVA
Diagnostics
Model Graphs
Analysis of Variance
Fit Statistics
Model Comparison Statistics
Coefficients
Coded Equation
Actual Equation
Model | 9191.78 | 1 | 9191.78 | 14.77 | 0.0006 | significant
C-Temp | 9191.78 | 1 | 9191.78 | 14.77 | 0.0006
Residual | 17424.45 | 28 | 622.30
Lack of Fit | 17269.83 | 26 | 664.22 | 8.59 | 0.1094 | not significant
Pure Error | 154.61 | 2 | 77.31
Cor Total | 26616.23 | 29
These rows were ignored for this analysis:
29, 25, 22, 21
Factor coding is Coded.
Sum of squares is Type III - Partial
The Model F-value of 14.77 implies the model is significant. There is only a 0.06% chance that an F-value this large could occur due to noise.
P-values less than 0.0500 indicate model terms are significant. In this case C is a significant model term. Values greater than 0.1000 indicate the model terms are not significant. If there are many insignificant model terms (not counting those required to support hierarchy), model reduction may improve your model.
The Lack of Fit F-value of 8.59 implies the Lack of Fit is not significant relative to the pure error. There is a 10.94% chance that a Lack of Fit F-value this large could occur due to noise. Non-significant lack of fit is good -- we want the model to fit.
For Help, press F1

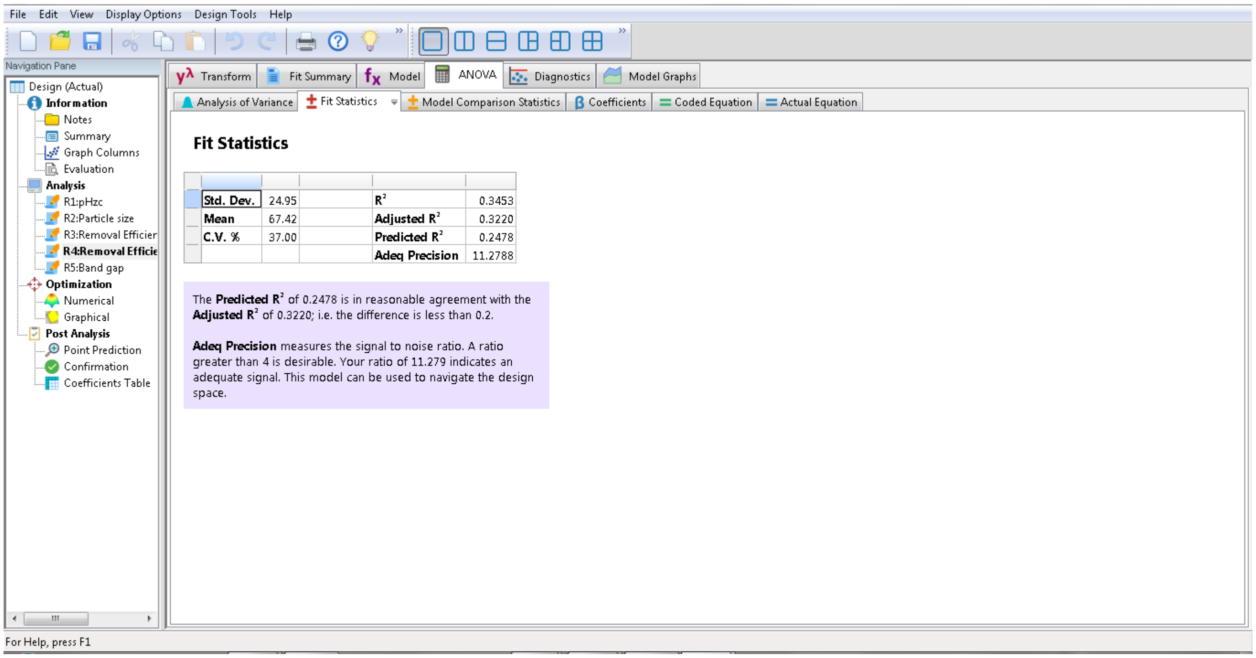
File Edit View Display Options Design Tools Help
Navigation Pane
Design (Actual)
Information
Notes
Summary
Graph Columns
Evaluation
Analysis
R1:pHzc
R2:Particle size
R3:Removal Efficier
R4:Removal Efficie
R5:Band gap
Optimization
Numerical
Graphical
Post Analysis
Point Prediction
Confirmation
Coefficients Table
Transform
Fit Summary
Model
ANOVA
Diagnostics
Model Graphs
Analysis of Variance
Fit Statistics
Model Comparison Statistics
Coefficients
Coded Equation
Actual Equation
Fit Statistics
Std. Dev. 24.95
Mean 67.42
C.V. % 37.00
R² 0.3453
Adjusted R² 0.3220
Predicted R² 0.2478
Adeq Precision 11.2788
The Predicted R² of 0.2478 is in reasonable agreement with the Adjusted R² of 0.3220; i.e. the difference is less than 0.2.
Adeq Precision measures the signal to noise ratio. A ratio greater than 4 is desirable. Your ratio of 11.279 indicates an adequate signal. This model can be used to navigate the design space.
For Help, press F1
